# Structural acoustic design of a sonicator to enhance energy transfer efficiency

**DOI:** 10.1016/j.ultsonch.2024.106804

**Published:** 2024-02-10

**Authors:** Sara Maghami, Örjan Johansson

**Affiliations:** Engineering Acoustics, Department of Civil, Environmental and Natural Resources, Luleå University of Technology, Luleå, Sweden

**Keywords:** Sonicator design, Energy transfer efficiency, Impedance matching, Parasitic modes, Bubbly liquid

## Abstract

The study focuses on developing a comprehensive design approach for a flow-through ultrasonic reactor (sonicator) to tackle challenges like low energy transfer efficiency and unstable system performance. The simulation accounts for structural vibrations, structural-fluid interactions, and pressure distributions within the cavitation zone under single-frequency excitation. Different geometrical designs of cylindrical sonicators are analyzed, with input parameters tailored to acquire higher acoustic cavitation intensity. The findings reveal a novel hexagonal ring-shaped excitation structure that reduces coupling losses, ensures uniform acoustic pressure distribution, and generates symmetric vibration mode shapes. The study emphasizes the separation of parasitic modes from the desired resonance frequency response and simulates the influence of bubbly liquid properties through complex wave numbers and harmonic responses. Experimental validation on a manufactured prototype, including mechanical and electrical impedance, sound pressure spectrum, and cavitation intensity, supports the simulated results. Ultimately, the sonicator exhibits three feasible resonance frequencies to be used pairwise at the certain temperature and input power interval for different applications.

## Highlights

• **Challenges in Adoption** of sonochemistry is restricted by industrial demands and economic feasibility, due to complex interplay of variables and up-scaling.
• **Multiphysical simulation tools** manage the interplay between piezo electric excitation and the wave propagation in structures and bubbly liquid.
• **Efficient Energy Transfer** is achieved by optimization of the cylindrical sonicator structure, hexagonal rings, and properties of bubbly water.
• **Dual Frequency Excitation** enhances efficiency and flexibility in sonochemical applications, in this case achieved by 18 uniform-size actuators.

## 1 Introduction

Ultrasound-induced cavitation has gained significant attention in advanced technology due to its physical and chemical effects [1], [2], [3], which offers the potential to surface decontamination, alleviating the negative environmental and health concerns associated with antimicrobial substances and preservation methods [4], [5], [6], [7], [8]. The possibility to operate a sonicator around (50 °C), causes less damage to proteins and bio-active properties which are sensitive to heat [9]. During the process of planting to harvest of fruits, various pesticides have been implemented, which in some cases remains in the surface of products even after home washing [10]. Ultrasound induced surface cleaning takes advantage of the acoustic streaming and micro-jet near the surface of a substrate to dislodge the foreign contaminants which are clung to the objects [11], [12], [13]. However, industries deal with challenges regarding energy efficiency, up-scaling, and the inherent multidisciplinary of sonochemical processing. The main issue appeals the design of large energy-efficient transforming geometries at high output power that fulfill the technical requirements in practical applications, especially where the process needs to be economically effective [14], [15], [16].

Sonoreactor modelling is of great interest for improving the cavitation efficiency considering aspects e.g., structural acoustics, sonochemistry, fluid mechanics, electronics, and material science [17], [18], [19]. Different reactor types [20], [21], designs, and operational parameters [22], [23] have been investigated over the years. The performance of a sonoreactor relies on various factors, including design, operating frequencies, and the properties of the medium [2]. The generation of ultrasonic waves through the piezoelectric effect introduces electrical losses, and additional losses arise from factors like coupling, damping, and heat. Resonances within the structure and fluid volume further influence the system. Factors such as flow characteristics, the presence of bubbles [24], and their size impact the distribution of ultrasound in the liquid. It’s important to note that bubbles can introduce significant variability and attenuation in the process. Thus, when working with sonoreactors, one can anticipate losses throughout the process [15], [16].

The present work endeavors to optimize the design parameters of a flowthrough sonicator for energy-efficient transfer of the electric input signals to transient cavitation events. The objective is to evaluate a flow-through sonicator concept in juice production, particularly in fruit cleaning and juice pasteurization. The design process employs multi-physical simulation to handle coupled modes and impedance matching, optimize radiation efficiency and provide an efficient energy transfer to the cavitation zone. Additionally, particular attention is given to both parasitic and harmonically related vibration modes in the structure. Other crucial consideration includes the design of electro-acoustic excitation structures and the assessment of propagation losses within the cylindrical sonicator when utilized a bubbly liquid.

## 2 Methodology

**Fig. 1:**
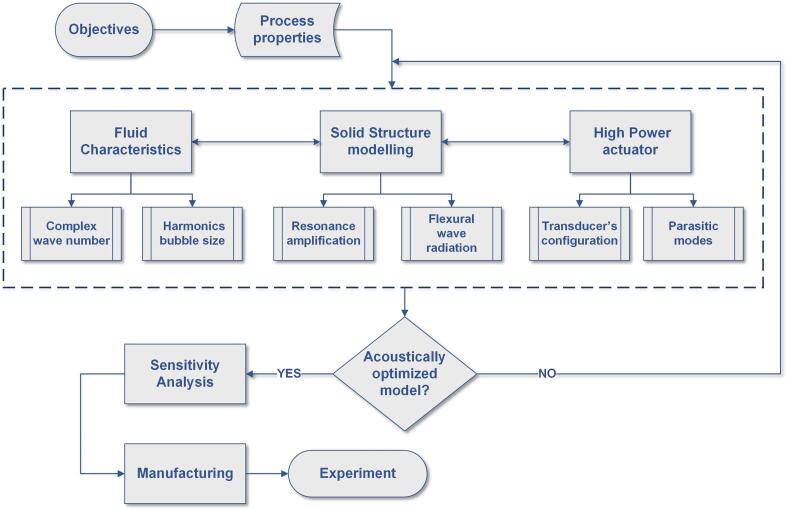
Design methodology for simulation and acoustic optimization of the sonicator.

Fig. 1 outlines the developed methodology for the cylindrical sonicator design. The multiphysical simulation procedures comprise solid structure modeling, high-power actuator design, and characterization of the bubbly fluid.

### 2.1 Structural design

Efficient energy transfer requires a strong coupling of specific types of vibration patterns and acoustic modes in the cavitation zone [25], [26], [27], [28], providing constructive interference within the sonicator [29], [30].

In a solid fluid coupled system, the vibration modes behave like that of a hollow shell or like a fluid column with a rigid wall in the attenuating region [29], [31]. The most efficient solid–fluid interactions give an emphasis on flexural modes. The same magnitude of transverse impedance of structure carrying bending wave and sound waves in fluid, can facilitate the exchange energy between the two media [32]. The approximated natural frequency of fluid-loaded structure can be estimated as:

(1) $$\omega_{m}≃\omega_{m}^{\prime }{\left(1+\rho_{o}/mk_{m}\right)}^{1/2}$$

where *ω'*_m_ is the natural frequency in a vacuum state, k_m_ the primary effective modal wave number of the vibration, *ρ*_0_ fluid density, m the average structural mass per unit area. It can be concluded from Eq. (1) that fluid loading normally affects the natural frequencies of the very low-order modes of a structure.

The target resonance frequency of the system is in the vicinity of or above critical frequency (intersection point in phase speed dispersion curve). For the frequency-dependent bending wave speed (c_B_), the eigen-frequency of a thin wall cylindrical structure with finite dimensions can be defined by Eq. (2):

(2) $$f_{s,l_{s}}=c_{B}\sqrt{{\left(\frac{s}{\pi D_{m}}\right)}^{2}+{\left(\frac{L}{2L_{s}}\right)}^{2}}$$

where s is an integer number of wavelength, l_s_ represents an integer number of half wavelength, D_m_ is the mean diameter of the cylinder, and L_s_ is the length of the cylinder. Eq. (3) defines the eigenfrequency of a fluid bounded by a rigid cylinder with open ends:

(3) $$f_{m,n,l_{f}}=\frac{c}{2}\sqrt{{\left(\frac{2\beta_{\mathrm{mn}}}{\pi D_{i}}\right)}^{2}+{\left(\frac{l_{f}}{L_{f}}\right)}^{2}}$$

where c is wave speed in fluid, D_i_ the inner diameter, L_f_ the length of the cylindrical fluid volume, l_f_ integer number of half wavelength, and *β* Bessel function. An important aspect is that the breathing mode of the structure is strongly coupled to the fluid column inside the cylinder. To achieve strong coupling between the structure and the fluid, the breathing mode frequency should be less than the radial cross-sectional eigenfrequency of the enclosed fluid [33].

The design starts by determining the fluid column’s natural frequency in a rigid cylinder, with emphasis on the radial mode shapes. According to Eq. (3), radial mode of fluid for the specific inner diameter and mode numbers (e.g., m_0_, n_2_) giving the target frequency at which two independent modes of pure axial and pure radial co-exist. Constructive interference of these modes can provide maximum amplification. The target eigenmodes of the cylindrical structure with a specific length are defined by three-dimensional finite element modeling. To provide high radiation efficiency, high radiation resistance is required. Three aspects of importance are: Cushioning effect; Bending wavelength; and Geometrical focusing effect.

The hexagonal designs have been proven to be efficient in the generation of cavitation intensity in case of cylindrical structures [34]. It also provides the possibility of combining bending and longitudinal mode shapes that gives good coupling and focusing effects on the contained fluid [33]. Hence, the circumferential mode number m_6_ was set which also keeps the bending wavelength of tube equal or greater than the acoustic wavelength for the tube diameter of 120 mm (biggest apple size in market). Hexagonal design also provides the possibility of combining the triangular geometry for double frequency excitation. The mode number m_6_ was defined such that f_6,0_ *<* f_fluid 2,0_ to reinforce the actuators’ vibration on the circumference.

The installation of the actuators and the flange design of the cylindrical structure are of crucial importance to ensure an energy efficient and resilient operational conditions of the sonicator.

### 2.2 Actuator design

Extensive research has focused on developing energy-efficient ultrasonic actuators, emphasizing increased vibration amplitude, boosting acoustic output power, improved electro-acoustic efficiency, and reduced temperature elevation [37], [38] to prevent operational failures [39], [40], [41], [42].

Incorporating differently shaped ultrasonic horns with a common actuator enhances out-of-plane displacement in structures [43], [44]. Fluid applications require wider vibrating surfaces and to maintain high acoustic power capacity the radiation patterns and impedance matching need to be controlled [14], [43]. The radiating element of the excitation structure can be of various shape to fulfill the criteria of strong coupling, optimize energy transfer efficiency, and enable a high intensity.

Sonotrodes are a commonly used design where the horn tip is inserted into the cavitation fluid. However, one issue is the horn tip erosion caused by high acoustic intensity, which may lead to unstable cavitation activity and imposes contamination within the liquid. The latter is of special concern in case of food applications [9]. Moreover, maintaining temperature control is crucial because active frequency variation can shift the positions of standing wave nodal areas, thereby reducing sonochemical efficiency.

In flow-through applications, the fluid is often enclosed by horn structures e.g., parallel plates or cylindrical structures. In such a case, the surface area between vibrating structure and the fluid is large compared to the size of the actuator(s). The cylindrical horn structure has the benefits of large radiating area, efficient couplings, and a geometrical focusing effect. despite the benefits of a cylindrical shaped horn structure, the interface between the actuator and the cylinder is critical and crucial for the cavitation efficiency [45].

Also, avoiding parasitic modes near resonance vibration modes is essential to prevent material fatigue. Bending mode shapes should not coincide with operating frequencies to prevent structure fatigue and energy loss. Longitudinal bolt vibrations should occur above the operating frequency to maintain clamping force at high power, with various bolt head positions available [46], [47].

**Fig. 2:**
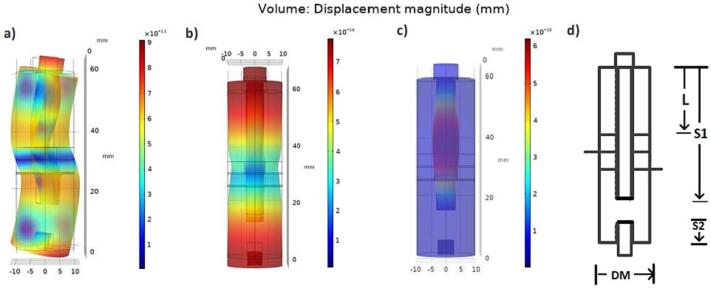
A and c) identified parasitic modes nearby the resonance frequency b) resonance frequency, d) design parameters.

Fig. 2 shows the actuator design principle highlighting the free length of the clamping bolt (S1), which is adjusted to avoid parasitic modes close to the resonance frequency. The free length between the actuator and the hexagonal ring (S2) is required to secure a resilient connection.

### 2.3 Sound propagation in a bubbly liquid

High power input to a liquid generates cavitation bubbles, which have a significant impact on sound wave speed and attenuation [48], [24], [45]. In a bubbly liquid, the wave speed is influenced by both the angular frequency and its complex value, which characterizes the attenuation of wave propagation. Near bubble resonance, the sound speed undergoes significant changes. This alteration is attributable to the oscillation of bubbles and the scattering of sound, ultimately resulting in an increase in sound attenuation due to power dissipation [49].

Several numerical acoustic pressure models of the bubbly liquid have been developed and verified in pilot-scale experiments. In this study, the mono disperse model by Commander and Prosperetti [50] was used. This model showed good agreement with predicting anti-node locations in bubble density up to n = 1*10^10^ m^−3^, while for acoustic pressure magnitude, the model showed slightly higher magnitude than the nonlinear Helmholtz model. This model performs well if the bubble fraction is low (1–2 %).

The general Helmholtz equation with a nonlinear term for the effect of the bubble attenuation was adapted which is defined by a complex wave number. The imaginary part of the wave number denotes the attenuation coefficient defining the amount of acoustic energy that is dissipated as damping in the medium. This attenuation has various origins e.g., volume fraction of bubbles [51].

The presented findings [52] indicate that the average measured bubble diameter is considerably smaller than the size associated with the fundamental resonance frequency. For instance, at a 20 kHz excitation frequency, the average bubble size is around 280 µm. However, in high-intensity ultrasound environments, bubbles tend to have sizes in the range of 10 to 7.5 µm, aligning with the 15th harmonic of the excitation frequency. In such conditions, bubbles resonating at the exact excitation frequency, or its close harmonics tend to implode rapidly and reduce in size. Occasionally, they may expand into larger gas pockets, which can negatively impact cavitation efficiency.

Table 1 summarizes the parameter values used in the simulation of the bubbly liquid. With respect to the number of bubbles per unit volume, the approximated distance between each bubble is 66 times bigger than the bubble size, which implies no interaction between bubbles.

**Table 1:** Summary of the applied parameters in the model [49], [53], [54], [55].

Parameter	Symbol	Value	Unit
Excitation frequency	*f*	29,400	[Hz]
Density of water*	*ρ*	998	[kg/m^3^]
Surface tension of water*	*σ*	0.0725	[N/m]
Speed of sound in water*	c	1481	[m/s]
Thermal diffusivity of the gas*	D_g_	2.08e-5	[m^2^/s]
Viscosity of water*	*µ*	8.90e-4	[Pa.s]
Specific heat ratio of gas	*γ*	1.4	–
Ambient liquid pressure	P_liq_	1e5	[Pa]
Active bubble radius	ract	6.9	[µm]
Number of bubbles per unit volume	n_b_	1e10	–
Bubble volume fraction	*β*	1.4e-5	–

*Note*: 20 °C is applied to calculation.

Earlier studies demonstrate that the chaotic oscillation of cavitation bubbles enhances sonochemical reaction rates. Dual-frequency excitation plays a crucial role in inducing chaotic bubble behavior. The transition of cavitation bubbles from periodic to chaotic oscillation can be facilitated by increasing the second frequency, reducing pressure amplitude ratios, and maintaining a specific range of phase difference [56].

## 3 Results and discussion

### 3.1 Structural acoustic modelling

The resonance frequency of the cylindrical shaped sonicator (f_c_) where f_c_ ≤ f_R2,0_ was defined with the prerequisite of a symmetric mode shape f_z,6_. Aiming for six circumferential wavelengths determines the segment length to form the total tube length. Tuning the geometry with respect to material properties and fluid coupling, the local maximum of average sound pressure in the fluid volume was found at around 29 kHz. At this resonance frequency, the modal vibrations and fluid mode shape fulfilled the design target of even pressure distributions with a maximum along the center line of the enclosed water volume. The model is defined by 189,222 tetrahedral and 46,324 triangles elements.

**Fig. 3:**
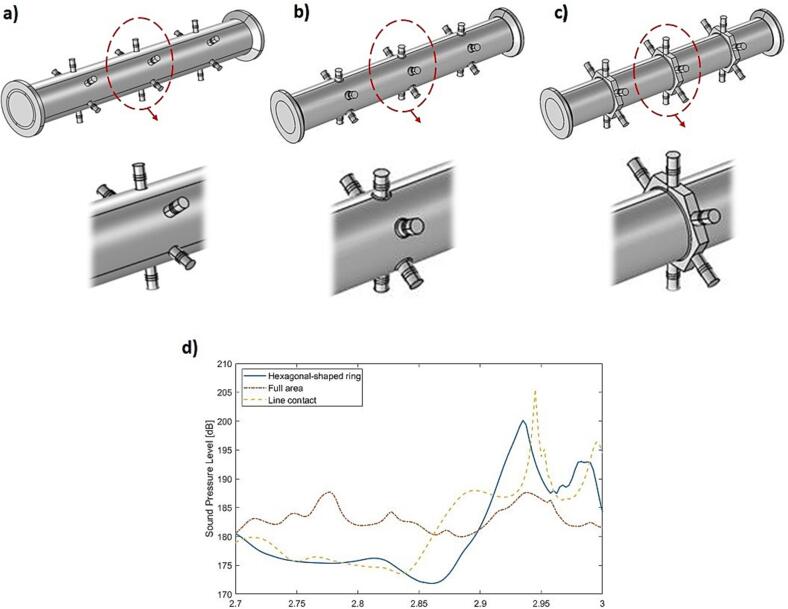
Various intersection design geometries of the ultrasonic sonicator: a) Line contact, b) Full area, c) Hexagonal-shaped ring, (d) Average SPL in the sonicated water volume with 18 sonotrodes.

Fig. 3 shows the three evaluated connection alternatives for connecting the actuators to the cylindrical tube wall. The simulation results indicates that a line contact between the actuator and the wall gives the best response. However, the hexagonal ring connection provides a more robust design, gives a better coupling between actuators and the cylindrical structure, and provides a more even vibration amplitude distribution. The six full wavelengths around the hexagonal ring enable minimum impedance and maximum amplification at the location of the actuators.

The effects on sound pressure and vibration mode shapes in relation to the geometry of the hexagonal ring have been evaluated related to the inner radius (IR = 60.5 to 62 mm), height (H = 95 to 115 mm), width (W = 20 to 30 mm), and rounded nut-shaped geometry. Altogether 16 different combinations were evaluated. Key factors were the symmetric circumferential modal behavior (m = 6), maximum parasitic modes separation, and a target frequency less than the fluid radial mode n_2_. To prevent the excitation of unwanted modes, preference is given to the separation of adjacent modes. The best result was achieved with the hexagonal-shaped ring parameters set to’Height = 110.0 mm, Width = 25.0 mm, Inner Radius = 61.25 mm’ (including the wall thickness).

The actuator design is critical to get a resonance frequency close to the sonicator operating frequency. The free resonance frequency of the actuator used is defined by the material properties of the clamping masses, and their length, the geometry and type of piezo material, and the clamping force provided by the connecting bolt. The connection bolt plays an important role with respect to fatigue and efficient energy transfer. The free length h of the pre-loaded bolt was optimized using parametric sweep eigen frequency modules in Comsol Multi-physics.

### 3.2 Acoustic optimization

To generate a higher and even distributed sound pressure in the water volume two more hexagonal rings, at a distance of an even number of axial bending wavelength (e.g., 6*λ*), were added. The final adjustment to reach the best possible response was made by modifying the length of the outer tube ends, wall thickness (h), and actuator frequency. After conducting a sensitivity analysis, trying 72* different combinations, the design targets were fulfilled at L = 998 mm, ID = 120 mm, h = 8.5 mm, and D_HR_ = 273.5 mm (distances between the hexagonal rings).

**Fig. 4:**
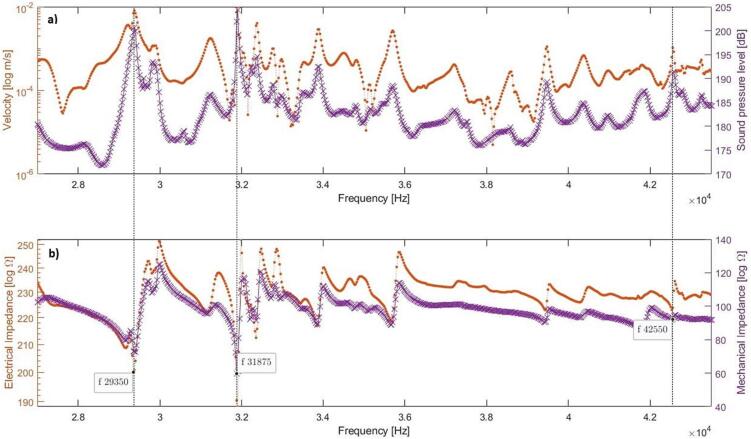
Simulated linear response of the sonicator excited by 18 actuators (N18, L25.3). a) Average SPL and vibration velocity on the interface between the actuator and hexagonal ring, b) The mechanical and electrical impedances.

Fig. 4 shows the simulated frequency response of the averaged sound pressure within the sonicator, the vibration velocity of the actuators, and the electrical and mechanical impedance when excited by 18 actuators. The target operational frequencies are defined by pressure maxima and corresponding impedance minima. The equivalence in minima and maxima locations between mechanical and electrical impedance shapes, despite varying magnitudes, suggests the potential for employing electrical impedance measurements to evaluate system behavior during processing. Fig. 4. indicates three resonances of interest at 29350, 31850, and 42550 Hz. However, the selection of the excitation frequency depends also on the pressure distribution within the water volume inside the sonicator as well as the vibration behavior of actuators.

**Fig. 5:**
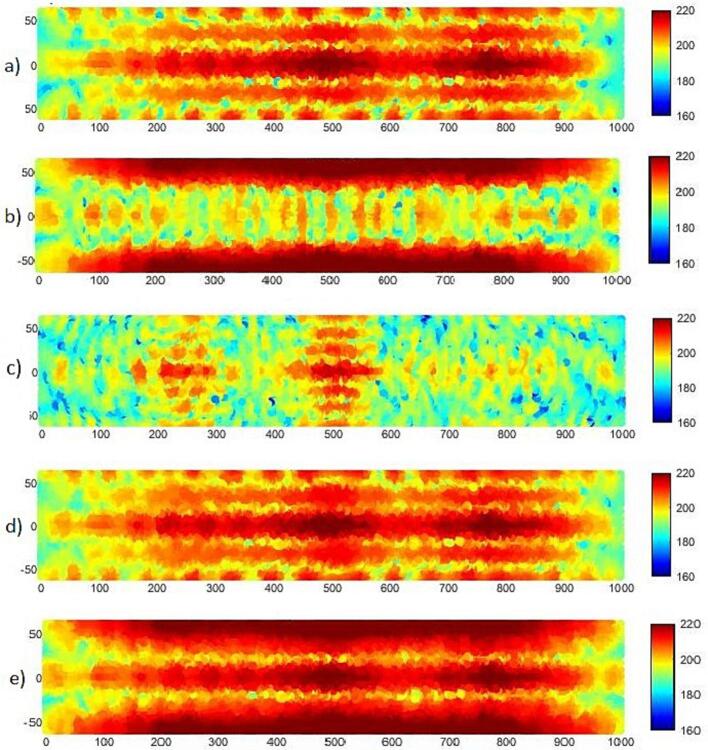
Sound pressure distribution in the cross-section of the fluid a) 29350 Hz, b) 31875 Hz, c) 42550 Hz, d) the superposition of the two modes a and c, e) the superposition of the two modes a and b.

Fig. 5 illustrates the maximum positive interference of the candidate frequencies. In the present application two different sound pressure distributions are of interest. For surface cleaning of apples an even pressure distribution within the volume is favorable, see Fig. 5. In case of pasteurization, high sound pressure along the central part of the volume is of interest. In such case, a poly-carbonate tube will be installed co-axially of the stainless-steel tube [57] to keep the apple juice in the high intensity zone, and to avoid metallic contamination of the juice.

The target mode shape for structural vibration and sound pressure distribution is achieved at 29350 Hz. This configuration entails all sonotrodes vibrating in phase along the axial direction, resulting in a uniform distribution of vibration across the cylinder wall. The bending wavelength is longer than the acoustic wavelength and gives an efficient excitation of the target acoustic mode with a focused pressure distribution along the cylinder axis.

The sonicator performance depends on excitation frequency, actuator vibration amplitude and signal types e.g., pulsed, swept, and bi-modal signals [35]. Signal characteristics like frequency bandwidth, sweeping rate, and monotonic direction play an essential role for the efficient growth and collapse of cavitation bubbles [36].

### 3.3 Modelling the effect of bubbly liquid

**Fig. 6:**
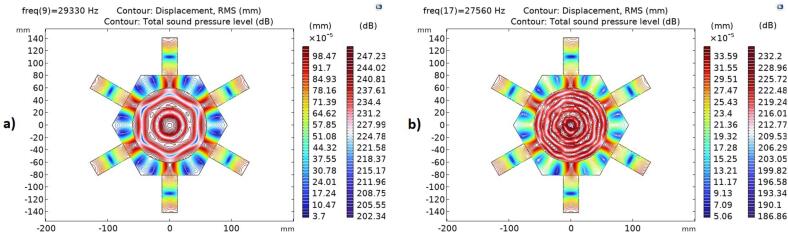
Contour plot of appropriate mode shapes in a: pure water, b: bubbly water.

Fig. 6 reveals the target vibration modes both in pure and bubbly liquid. Although displacements are suppressed down to one-third in the case of bubbly liquid, the structural vibration modes are similar for both cases. The explanation relates to the reduced wave speed in bubbly liquid at high pressure e.g., 700 m/s when bubble sizes relate to the 15th harmonic (T = 20C). In this case a new resonant condition occurs where the resonant radial acoustic mode appear at a higher order number, n = 4. (n = 2 in pure water).

**Fig. 7:**
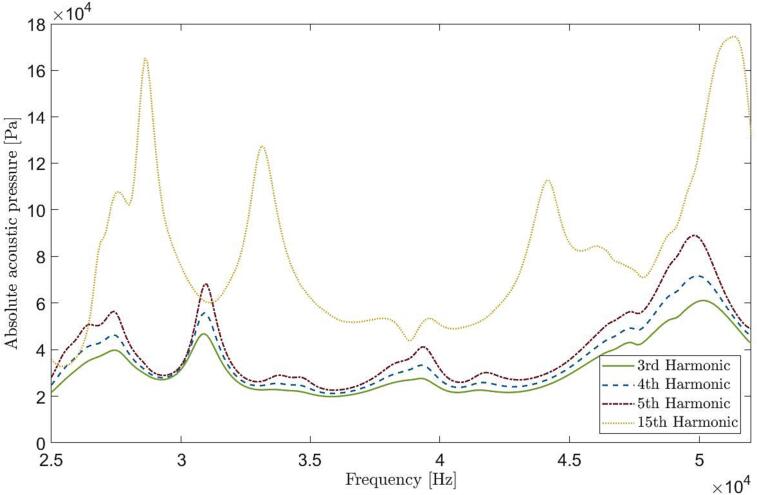
Volume average of absolute acoustic pressure in a bubbly liquid with bubble size related to different harmonics - N18, L25.3 (Bubble size of various harmonics 3rd, 4th, 5th, 15th is expressed respectively; 34, 25, 20, 6.9 [µm]).

Using a 300 V input voltage in simulating bubbly liquid properties produces a volume-averaged absolute acoustic pressure of 170 kPa. This leads to increased losses, reduced wave speed, and the suppression of pressure peaks associated with complex modes, although radial modes are less affected. Fig. 7 illustrates how different bubble sizes (corresponding to various harmonics) affect the pressure spectrum. Larger bubble sizes (lower harmonics) cause more pronounced sound wave attenuation due to an increase in the imaginary component of the complex wave number.

### 3.4 Experimental verification

**Fig. 9:**
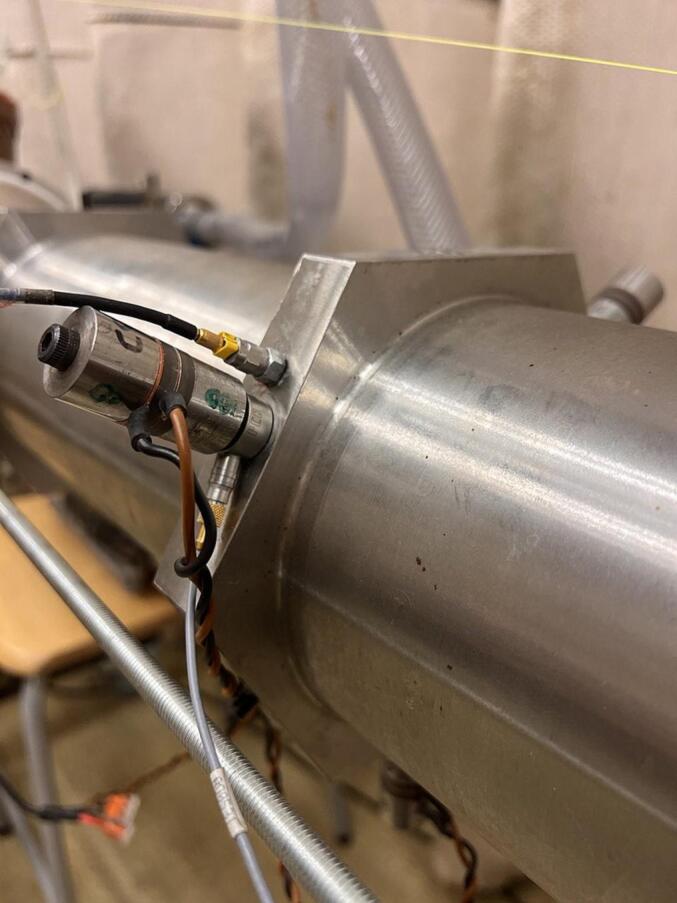
Mechanical point impedance measurement setup using three small actuators, all in combination with a force transducer between each actuator and the hexagonal ring.

Fig. 9 shows the experimental setup used for verification of the water filled sonicator structure with respect to acoustic pressure, and the mechanical and electrical impedance. To be able to determine the mechanical impedance in parallel to sound pressure distribution, measurements were performed using three small actuators attached to the mid-hexagonal ring (120degree separation). Force transducers,’Dytran PCB-201a’, were mounted in between the actuators and the hexagonal ring. The point impedance was determined by measurement of acceleration’PCB353M15′ at the hexagonal ring close to the force transducer. The sound pressure was measured using a pressure sensor (PCB 113B21) connected to a FFT-based measurement system (Bruel and Kjaer Connect). To ensure a good spatial average of the sound pressure, the measurement along the sonicator axis was replicated four times. In the case of simulated results, pressure has been determined as an average of a narrow volume (D = 10 mm) around the cylinder axis.

**Fig. 10:**
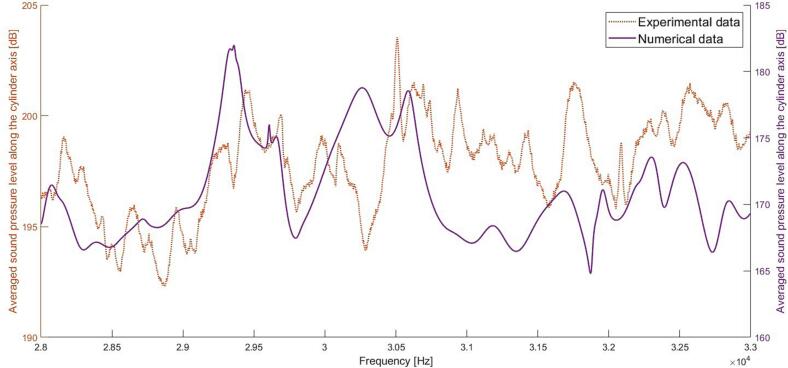
Simulated and measured averaged sound pressure level along the cylinder axis when excited by three small actuators (N3, L16).

Fig. 10 shows the simulated and measured pressure spectrum along the cylinder axis. The results show a similar trend with a downward frequency shift 1–2 % of the main peaks in the pressure spectrum. The measured spectra exhibit a higher level of detail but also an elevated noise floor, a phenomenon likely attributed to the employed averaging procedure and the utilization of a chirp signal. An additional noteworthy aspect is the observation of a nonlinear response induced by cavitation bubbles, despite the relatively low electrical input power of 10 W. The smoothness of the simulated spectrum can be attributed to the ideal geometry, the absence of cavitation bubbles, and a loss factor of 1 %. The loss factor used in the simulation was set based on results from electrical impedance experiments on another cylindrical structure at high power excitation.

**Fig. 11:**
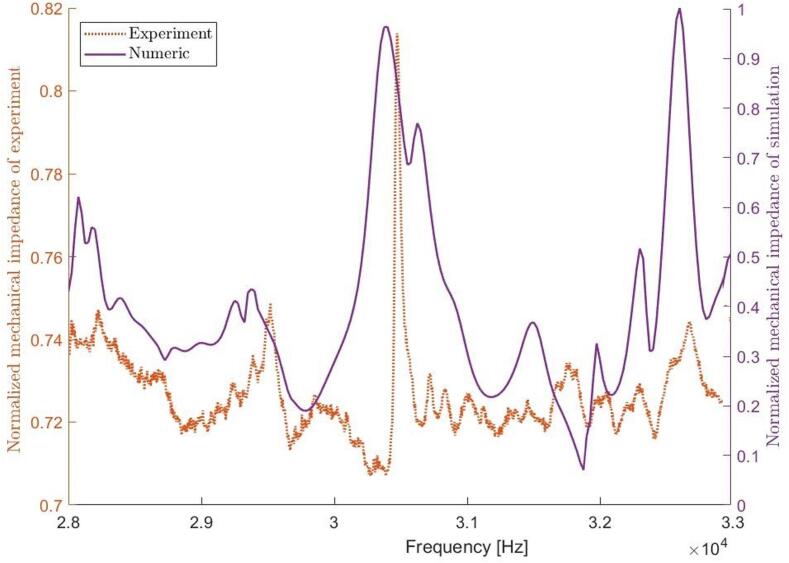
Normalized mechanical point impedance, comparison between experiment and numeric simulation excited by three small actuators (N3, L16).

Fig. 11 shows the simulated and measured mechanical impedance of the water-filled tube. A 1 % shift of the resonance frequencies can be observed, which is like the deviations between sound pressure spectra. There are partial similarities between spectra but the simulated impedance minima at 31.8 kHz is related to an attenuated response in the simulated pressure spectrum. The reason is likely linked to the expected mode shape with a high pressure along the cylinder wall (Fig. 5b) causing a cushioning effect. The strong coupling between the electrical and mechanical impedance of the sonicator seen in the simulations (Fig. 4b) is experimentally verified. The coupling between acoustic response and electrical impedance provides an efficient control of the sonicator’s cavitation performance.

The experimental verification of the sonicator with 18 actuators was made at various electrical power levels. The performed measurements regarding foil test were made at 360 W, and the electrical impedance (CLIO v12, Live transfer function, chirp signal, 192 kHz sampling frequency) was determined at 0.2 and 20 W. The simulation with bubbly liquid gives an electrical impedance minimum for the target resonance at 28.5 kHz, which is 1000 Hz higher than the corresponding measured impedance minimum at 27.5 kHz. There is also a good agreement at the corresponding anti-resonance at 29.0/29.3 kHz. The lack of detail in the simulated electrical impedance below 28 kHz and above 34 kHz relates to the bubbly liquid properties, which were defined based on the pressure distribution around the target resonance. However, the unified general trend shows some similarities except for the attenuation of 31.8 kHz. The attenuated resonances above 30 kHz is linked to the changes in the speed of sound, and losses in the bubbly liquid (Fig. 5b). These findings were also confirmed during high power measurements and likely related to a weak coupling due to an extensive bubble generation in the boundary region.

**Fig. 8:**
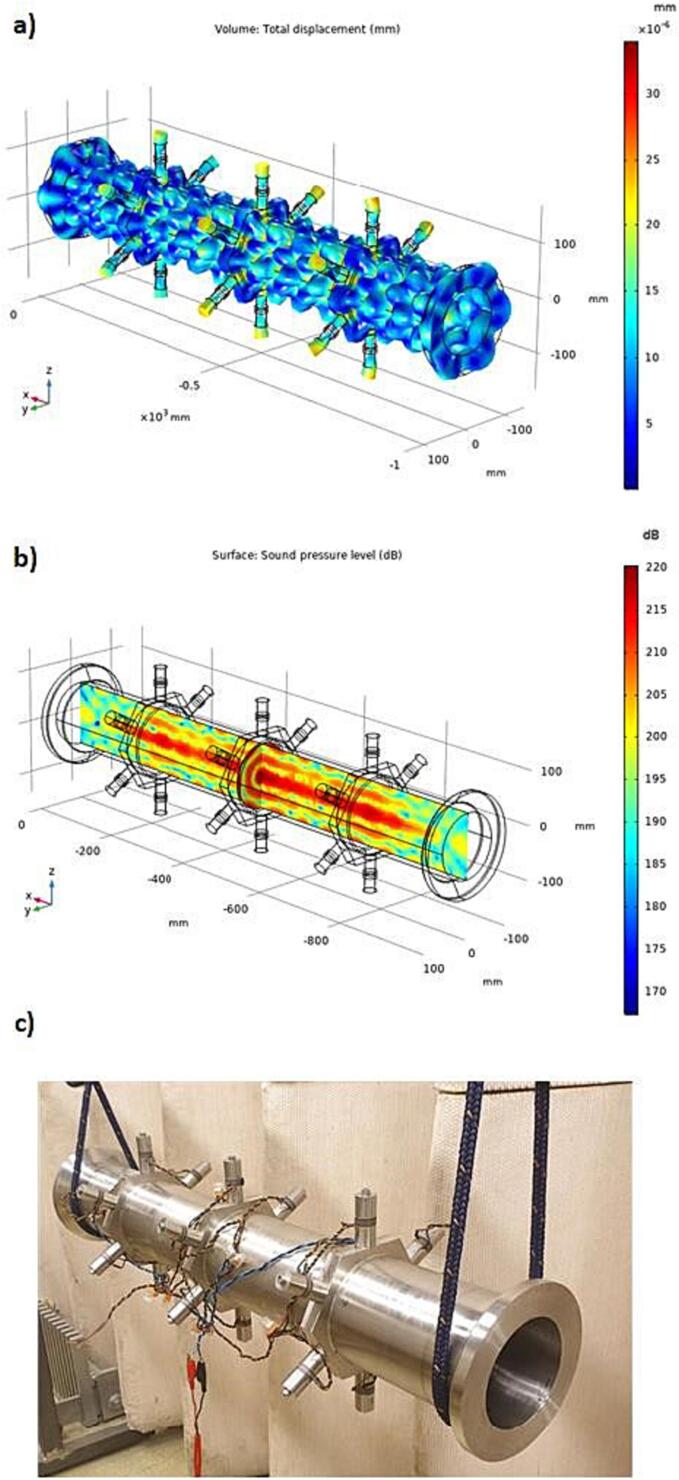
Optimized design for pure water at the resonance frequency 29.3 kHz a) Simulated vibration displacement mode shape [mm], b) Simulated sound pressure level distribution [dB], c) Manufactured prototype.

**Fig. 12:**
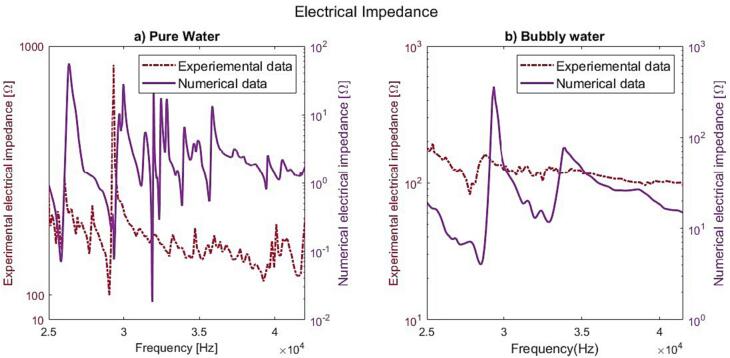
Simulated and measured electrical impedance: a)pure water, b)bubbly water, when excited by 18 actuators (L25.3) at 100 W input power.

The sonicator concept was validated by excitation using 18 actuators of equal size (L25.3), see Fig. 8c. Fig. 12 shows the simulated and measured electrical impedance at two power levels. The low level (2mW) secures a linear response, and the high-power setting (20 W) generates a cavitation response. In pure water, the simulated impedance minimum for the target mode occurs at 29.3 kHz (Fig. 8), and measured minimum is at 29.0 kHz. In case of bubbly liquid, the simulated minimum impedance is at 28.5 kHz and experimentally verified at 27.8 kHz. The reduction in resonance frequency is due to the increased loss caused by cavitation and the reduced wave speed. The simulation shows that the target pressure distribution is fulfilled at both 27,560 and 28600 Hz, however, the target vibration mode shape is only fulfilled at 27560 Hz, see Fig. 6b.

**Fig. 13:**
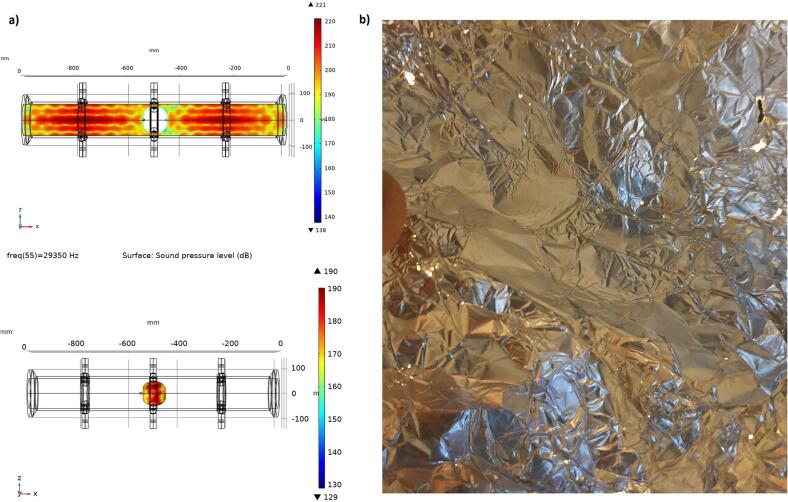
A) sound pressure level distribution inside the sonicator with an apple added. b) Erosion pattern on a foil wrapped around an apple flowing through the sonicator.

Fig. 13 illustrates the pressure distribution with an apple inside the sonicator. The pressure distribution over the apple surface is sufficient to generate cavitation. The cavitation intensity was validated by the erosion of an aluminum foil wrapped around the apple flowing through the sonicator (20 s, T = 35C, 360 W). At an input power of 360 W, the target resonance frequency reduces to 27.0 kHz and the impedance is around 480 Ω for 18 actuators in parallel. However, the most energy efficient operating conditions require control of both water temperature and input power since the physical properties of a bubbly liquid at atmospheric conditions depends on both the input power and temperature.

## 4 Conclusion

Sonochemistry has been of interest to the process industry for many years, but performance demands regarding robustness, energy efficiency, and economic feasibility have limited the use. The objective in this study was to develop and evaluate a multiphysical simulation procedure. The goal was to develop a sonicator for small scale industrial applications, enabling the generation of sufficiently high cavitation intensity in the flow-through zone for the cleaning of apples, and for pasteurization of apple juice. The design procedure involved coupled structural fluid interaction, piezoelectric actuator optimization, and effects on wave propagation by a bubbly liquid. The experimental verification of the sonicator shows good agreement at the target frequency. The sonicator generates intensive cavitation effects at three frequencies, with appropriate sound pressure distributions and vibration mode shapes. The best response is at 29 kHz when excited by 18 optimized piezoelectric actuators connected to the cylindrical structure by three hexagonal rings. The ring geometry and the positions have been optimized to achieve even distributed vibrations that enable efficient energy transfer and high cavitation intensity in the bubbly liquid of the flow through zone. Feasible results were achieved by avoiding parasitic modes close to the target resonances of the sonicator. One important aspect is that the physical properties of the fluid inside the sonicator changes due to cavitation. The reduction of wave speed shifts the target radial resonance to a higher radial mode number. Another effect is that losses within the bubbly liquid effectively suppress the more complex pressure modes. Peak frequencies of averaged sound pressure match perfectly with local electrical impedance minima, providing efficient monitoring and control of the sonicator performance. Dual frequency excitation of the sonicator gives flexibility and efficiency for different sonochemical applications and enhances performance. The use of 18 actuators of the same size gave better results in dual operation than the combination of long and short actuators (low and high frequency).

## CRediT authorship contribution statement

**Sara Maghami:** Data curation, Formal analysis, Investigation, Methodology, Software, Validation, Visualization, Writing – original draft, Writing – review & editing. **Örjan Johansson:** Conceptualization, Data curation, Formal analysis, Funding acquisition, Investigation, Methodology, Project administration, Resources, Supervision, Validation, Writing – review & editing.

## Declaration of competing interest

The authors declare that they have no known competing financial interests or personal relationships that could have appeared to influence the work reported in this paper.

## Data Availability

Data will be made available on request.
